# Supplementary material for: Microtubules regulate pancreatic β-cell heterogeneity via spatiotemporal control of insulin secretion hot spots
Source: eLife. 2021 Nov 16;10:e59912. doi: 10.7554/eLife.59912 (PMC8635970; doi:10.7554/eLife.59912)
Supplement: Figure 2—source data 2. — Script is annotated. [file elife-59912-fig2-data2.pdf]

Matlab script for identification of secretion events and clusters in cells:

```
% Number of cells per movie as identified by ROIs saved as .csv files
fnr=sprintf('*.csv');
Files=dir(fnr);
Nfile=length(Files); roi_number = Nfile;
% Create matrix where we will put counts for each roi
Total_counts = zeros(roi_number,2);
% Numbering count matrix
for i=1:roi_number
    Total_counts(i,1)= i;
end

% Reading the coordiantes of the secretion events identified in ImageJ
Points=xlsread('Noc High 11-6 Plate 3.xlsx');
time1 = Points(:,1);
col1 = Points(:, 4);
col2 = Points(:, 5);
Points = [col1 col2];

%This will be used to keep track of what cell ROI each secretion event is in
loc = zeros(size(col1,1),1);

clf
%Round the points to an integer
Points_rounded = round(Points);
%Densitiy-based scanning for clusters- 9 pixels (1.44um) was the search
%radius, 3 points is the minimum number of events that can be defined as a
%cluster
idx = dbscan(Points, 9, 3);
%This gives the number of clusters present
No_clusters = max(idx);
Cluster_sizes = zeros(No_clusters, 2);
for u=1:No_clusters
  Cluster_sizes(u,1) = u;
  A = find(idx(:,1)==u);
  Cluster_sizes(u,2)=size(A,1);
end


% This creates a matrix of the rounded points with the cluster label in the
% first column
Cluster_points = [idx Points_rounded];
%rows with '-1' in the first column are rows that are not in a cluster
indices = find(Cluster_points(:,1)==-1);
%deletes the rows that are not in a cluster
Cluster_points(indices,:) = [];
%Locate the unique cluster points
[~,idx1] = unique(Cluster_points(:,1));
Cluster_points = Cluster_points(idx1,:);
%Matrix with only one point to represent each cluster
```

```
Cluster_points(:,1) = [];

% Reading each ROI pixel coordinates of cells in loop
fnr=sprintf('*.csv');
Files=dir(fnr);
Nfile=length(Files);
for fileno=1:Nfile
  data = csvread(Files(fileno).name,1,0);
    x = data(:,1); y = data(:,2); k=boundary(x,y,1); plot(x(k),y(k),'black'); data=[x y];
    meanx = mean(x);meany=mean(y);
    Roi_number = string(fileno);
    text(meanx,meany,Roi_number);
    C = ismember(Points_rounded,data,'rows'); intersection_points = sum(C);


    loc(C) = fileno;
    Total_counts(fileno,2)= intersection_points;

B = ismember(Cluster_points,data,'rows'); cluster_number = sum(B);
Total_counts(fileno,3) = cluster_number;
hold on
end

Points_loc_time = [Points loc time1];

%Plot the points as clusters
a = [idx Points_loc_time];
a = sortrows(a,1);
Points_loc_time = a;
a = num2cell(a);
a(cell2mat(cellfun(@(elem) elem == -1, a(:, :), 'UniformOutput', false))) = {'No Clstr'};
a = string(a);

%creates a figure showing cell borders and secretion events either
%clustered or non clustered
fig = gscatter(Points_loc_time(:,2),Points_loc_time(:,3),a(:,1));
Non_cluster = fig(1); Non_cluster.Color = 'k';
hLeg = legend('fig');
set(hLeg,'visible','off');
xlim([0 500]);
ylim([0 500]);
hold off

Total_counts;
colNames = {'ROI_Number','Counts','Number_of_Clusters'};
sTable = array2table(Total_counts, 'VariableNames',colNames);
hold off
%Creation of three excel files with results
v = Points_loc_time; Points_loc_time(:,1)=v(:,5); Points_loc_time(:,5)=v(:,1);
Points_loc_time = sortrows(Points_loc_time,1);
xlswrite('time__centroid__roi__cluster-3.xlsx',Points_loc_time)
```

```
xlswrite('roi_number__counts__cluster_num-3.xlsx',Total_counts)
xlswrite('Sizes_of_clusters-3.xlsx',Cluster_sizes)
```
