# Supplementary material for: Microtubules regulate pancreatic β-cell heterogeneity via spatiotemporal control of insulin secretion hot spots
Source: eLife. 2021 Nov 16;10:e59912. doi: 10.7554/eLife.59912 (PMC8635970; doi:10.7554/eLife.59912)
Supplement: Figure 2—source data 3. — Script is annotated. [file elife-59912-fig2-data3.pdf]

```
%The purpose of this script is to count the number of events that occur within set
%Region of Interest (ROI) boundaries as well as detecting clustering among
%those events.

%Inputed into the script are ROI coordinates in the form of csv files as
%well as coordinates of events, also in the form of a csv file.

%Outputed from the script is a figure displaying the ROIs as well as the
%events with clusters color-coded. Also outputed is an excel sheet with
%information about the ROI counts and clusters.


clear all; clf;
%Insert name of Experiment and Results
Experiment_name = 'Correlative Microscopy';
Results_name = 'Results_g10pixel.csv';
Results_set_number = '10';

%Read this results file
Results = csvread(Results_name,1,0);
x_coord = Results(:,2); y_coord = Results(:,3); time = Results(:,4);
XY = [x_coord y_coord]; %Gives 2 column matrix of result coordinates
No_points = size(XY,1); %Gives number of points in the results

%Reading the ROI Coordinates
fnr = sprintf('*csv');
Files = dir(fnr);
ROI_number = length(Files)-1; %ONLY IF THE RESULTS FILE IS ALSO .CSV (as opposed to .
xls)
%Files(length(Files))=[]; %Deleting the results .csv from the ROI files

%Matrix for ROI_counts
ROI1_counts2_nonclustered3_clustered4_numclusters5 = zeros(ROI_number,5);
for i = 1:ROI_number
    ROI1_counts2_nonclustered3_clustered4_numclusters5(i,1)=i;
end

%This matrix will contain all information about each individual result
%point
T1_x2_y3_roi4_posc5 = [time x_coord y_coord zeros(No_points,1)];

%This finds clusters in the result points based on x and y coordinates
%dbscan(X,epsilon,minpts)
idx_position = dbscan(XY, 5, 3);
idx_position(idx_position==-1)=0; %Changing -1 to 0 for non-cluster points
%Input these index labels into the point matrix
T1_x2_y3_roi4_posc5 = [T1_x2_y3_roi4_posc5 idx_position];
No_clusters = max(idx_position); %This gives the number of clusters
```

```
%Creates a matrix with the sizes of each cluster
Cluster1_size2 = zeros(No_clusters,2);
for i=1:No_clusters
    Cluster1_size2(i,1)=i;
    A = find(T1_x2_y3_roi4_posc5(:,5)==i);
    Cluster1_size2(i,2)=size(A,1);
end

%Round point coordaintes to an integer to check if they are contained in
%ROI coordinates
Points_rounded = round(XY);
%Reading each ROI pixel coordiantes in a loop
loc = zeros(No_points,1);

for fileno=1:ROI_number
    ROI_file_name = append(num2str(fileno),'.csv');
    ROI = csvread(ROI_file_name,1,0);
    X_ROI = ROI(:,1); Y_ROI = ROI(:,2);
    k=boundary(X_ROI, Y_ROI,1); plot(X_ROI(k),Y_ROI(k),'black')
    ROI_coord = [X_ROI Y_ROI];
    %Plot number of ROI on the Figure
    meanx = mean(X_ROI); meany = mean(Y_ROI);
    text(meanx,meany,num2str(fileno));
    %Detecting if the event resides in the ROI
    C = ismember(Points_rounded,ROI_coord,'rows');
    intersection_points=sum(C); ROI1_counts2_nonclustered3_clustered4_numclusters5↙
(fileno,2) = intersection_points;
    loc(C)=fileno;
    hold on
end

ROI = csvread(Files(1).name,1,0);
 x = ROI(:,1); y = ROI(:,2); k=boundary(x,y,1); plot(x(k),y(k),'black'); ROI=[x y];
    meanx = mean(x);meany=mean(y);
    Roi_number = string(1);
    text(meanx,meany,Roi_number);

title(append(Experiment_name,' ',Results_set_number));
T1_x2_y3_roi4_posc5(:,4)=loc.';

%Sort rows based on clusters so the legend is in order
T1_x2_y3_roi4_posc5 = sortrows(T1_x2_y3_roi4_posc5,5);
%Naming clusters for the figure legend
a = T1_x2_y3_roi4_posc5(:,5);
a = num2cell(a);
a(cell2mat(cellfun(@(elem) elem == 0, a(:, :), 'UniformOutput', false))) = {'No↙
Clstr'};
a = string(a);
```

```
%Plotting the points based on cluster label (for color coordination)
fig = gscatter(T1_x2_y3_roi4_posc5(:,2),T1_x2_y3_roi4_posc5(:,3),a(:,1));
Non_cluster = fig(1); Non_cluster.Color = 'k'; %makes points black if not in a ↙
cluster
legend('Location','eastoutside');
axis equal;

hold off

%Find number of clustered and non-clustered events in each ROI
for i=1:ROI_number
    idx = T1_x2_y3_roi4_posc5(:,4)==i;
    a = T1_x2_y3_roi4_posc5(idx,:);
    if sum(idx) ~= 0
       ROI1_counts2_nonclustered3_clustered4_numclusters5(i,4)= sum(a(:,5)==0);
       ROI1_counts2_nonclustered3_clustered4_numclusters5(i,3)=sum(a(:,5)~=0);
    end
end

%Finding the number of unique clusters
Clusters = unique(T1_x2_y3_roi4_posc5(:,5)); Clusters = Clusters(Clusters ~= 0);
No_clusters = size(Clusters,1);
%Creates a matrix with the sizes and centers of each cluster
Cluster1_sizes2_center34 = zeros(No_clusters,4);
for i=1:No_clusters
    Cluster1_sizes2_center34(i,1) = i;
    A = find(T1_x2_y3_roi4_posc5(:,5)==i);
    Cluster1_sizes2_center34(i,2)=size(A,1);
end
z = zeros(No_clusters,2);
for m = 1:No_clusters
    for i = 1:No_points
        %Creating a column vector of each of the points in cluster 'm'
        idx = T1_x2_y3_roi4_posc5(:,5)==m;
        w = T1_x2_y3_roi4_posc5(idx,:);
        %Column vectors of the x and y coordinates of the points in the
        %clusters
        x = w(:,2); y = w(:,3);
        meanx = mean(x); meany = mean(y);
        Cluster1_sizes2_center34(m,3)=meanx;
        Cluster1_sizes2_center34(m,4)=meany;
        Cluster_number = string(m);
        %Adds label to each of the clusters on the figure
    end
end
%Finding which ROI the cluster occurs in
locC = zeros(No_clusters,1);
Cluster_location_rounded = [round(Cluster1_sizes2_center34(:,3)) round ↙
(Cluster1_sizes2_center34(:,4))];
```

```
for fileno=1:ROI_number
    ROI_file_name = append(num2str(fileno),'.csv');
    ROI = csvread(ROI_file_name,1,0);
    x = ROI(:,1); y = ROI(:,2); ROI=[x y];
    C = ismember(Cluster_location_rounded,ROI,'rows'); intersection_points = sum(C);
    locC(C) = fileno;
    ROI1_counts2_nonclustered3_clustered4_numclusters5(fileno,5)= ↙
intersection_points;
    hold on
end

set(findall(gcf,'-property','FontSize'),'FontSize',8);

%Writing exported excel file
Excel_export_name = append(Experiment_name,'-', Results_set_number,'-Exported Data.↙
xls');

col_header={'ROI Number', 'Total Counts','Cluster Counts','Non-Cluster↙
Counts','Number of Clusters'};
xlswrite(Excel_export_name,col_header,'Sheet1','A1');
xlswrite(Excel_export_name,↙
ROI1_counts2_nonclustered3_clustered4_numclusters5,'Sheet1','A2');

col_header = {'Time', 'Centroid X', 'Centroid Y', 'ROI', 'Cluster'};
xlswrite(Excel_export_name,col_header,'Sheet2','A1');
xlswrite(Excel_export_name,T1_x2_y3_roi4_posc5,'Sheet2','A2');

if No_clusters ~= 0
    col_header={'Cluster Number','Counts'};
    xlswrite(Excel_export_name,col_header,'Sheet3','A1');
    xlswrite(Excel_export_name,Cluster1_size2,'Sheet3','A2');
end
```
