# Supplementary figures and images for: Microtubules regulate pancreatic β-cell heterogeneity via spatiotemporal control of insulin secretion hot spots
Source: eLife. 2021 Nov 16;10:e59912. doi: 10.7554/eLife.59912 (PMC8635970; doi:10.7554/eLife.59912)

ControlLow

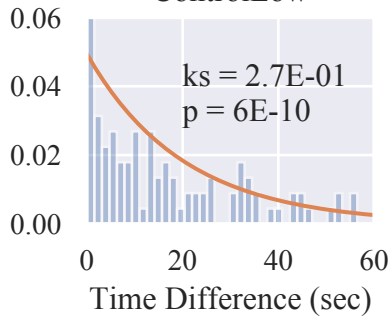

NOCLow

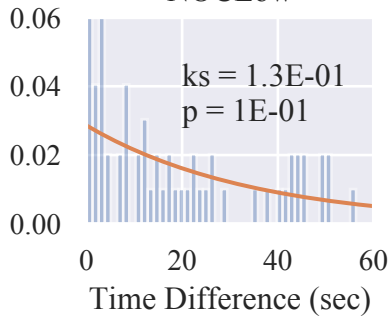

TaxolLow

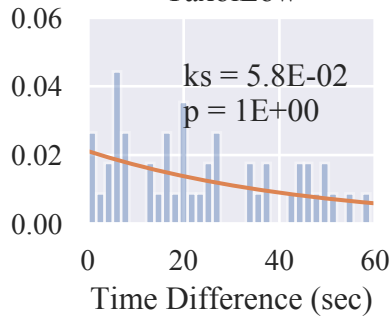

ControlHigh

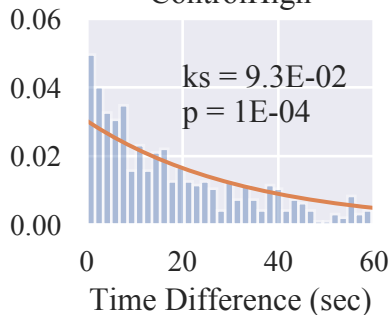

NOCHigh

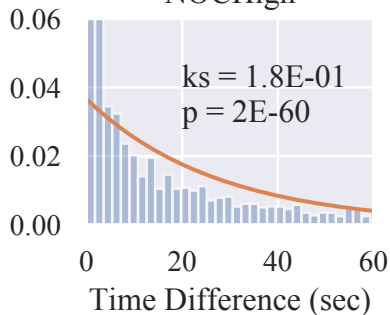

TaxolHigh

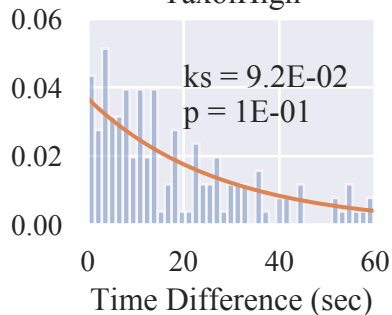

Supplement: Figure 5—source data 2. [file elife-59912-fig5-data2.zip › Figure 5-source data 1-Python scripts that produce statistical analysis and plots for Figure 5B-F/StatisticalAnalysis/Cluster_WaitTimes.pdf]

# 95.0% Credible Interval

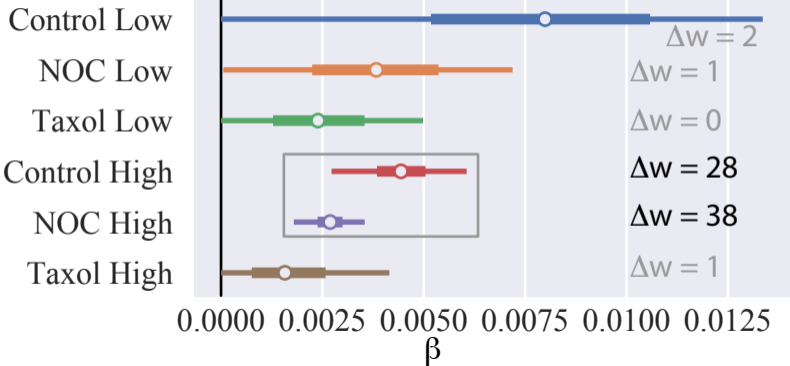

Supplement: Figure 5—source data 2. [file elife-59912-fig5-data2.zip › Figure 5-source data 1-Python scripts that produce statistical analysis and plots for Figure 5B-F/StatisticalAnalysis/ForestPlot_Beta_keep.pdf]

ControlLow

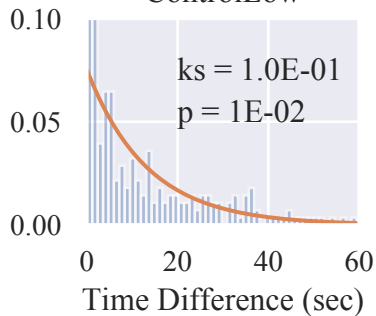

NOCLow

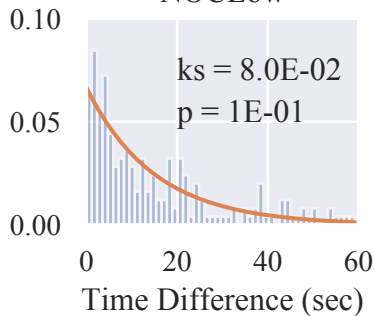

TaxolLow

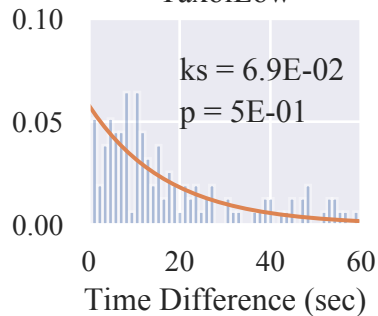

ControlHigh

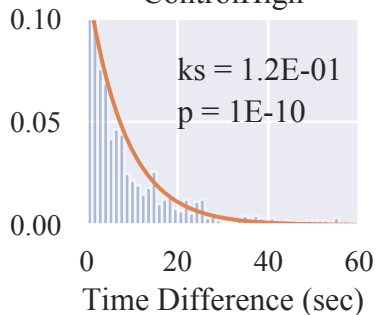

NOCHigh

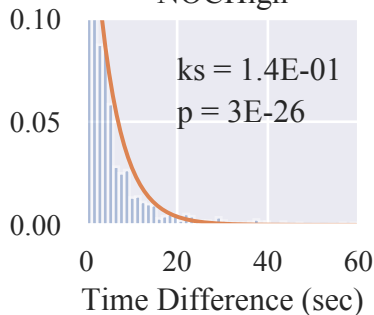

TaxolHigh

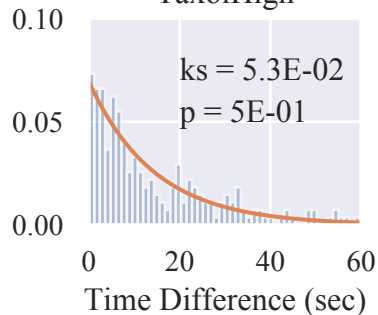

Supplement: Figure 5—source data 2. [file elife-59912-fig5-data2.zip › Figure 5-source data 1-Python scripts that produce statistical analysis and plots for Figure 5B-F/StatisticalAnalysis/NonCluster_WaitTimes.pdf]

KS P-value

$10^0$

$10^{-1}$

$10^{-2}$

3

4

5

6

7

8

9

10

Cluster Size

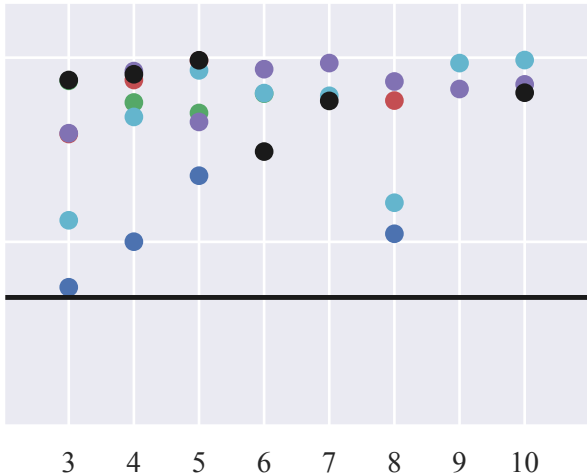

Supplement: Figure 5—source data 2. [file elife-59912-fig5-data2.zip › Figure 5-source data 1-Python scripts that produce statistical analysis and plots for Figure 5B-F/StatisticalAnalysis/WaitTime_vs_Size_Pvals.pdf]
