# Supplementary material for: Microtubules regulate pancreatic β-cell heterogeneity via spatiotemporal control of insulin secretion hot spots
Source: eLife. 2021 Nov 16;10:e59912. doi: 10.7554/eLife.59912 (PMC8635970; doi:10.7554/eLife.59912)
Supplement: Transparent reporting form [file elife-59912-transrepform1.pdf]

eLIFE
1st Floor
24 Hills Road
Cambridge CB2 1JP, UK
P 01223 855340
W elifesciences.org
T @elife

eLife's transparent reporting form

We encourage authors to provide detailed information *within their submission* to facilitate the interpretation and replication of experiments. Authors can upload supporting documentation to indicate the use of appropriate reporting guidelines for health-related research (see EQUATOR Network), life science research (see the BioSharing Information Resource), or the ARRIVE guidelines for reporting work involving animal research. Where applicable, authors should refer to any relevant reporting standards documents in this form.

If you have any questions, please consult our Journal Policies and/or contact us: editorial@elifesciences.org.

Sample-size estimation

- You should state whether an appropriate sample size was computed when the study was being designed
- You should state the statistical method of sample size computation and any required assumptions
- If no explicit power analysis was used, you should describe how you decided what sample (replicate) size (number) to use

Please outline where this information can be found within the submission (e.g., sections or figure legends), or explain why this information doesn't apply to your submission:

This information can be found in the material and methods, subsection Experimental Design.

Replicates

- You should report how often each experiment was performed
- You should include a definition of biological versus technical replication
- The data obtained should be provided and sufficient information should be provided to indicate the number of independent biological and/or technical replicates
- If you encountered any outliers, you should describe how these were handled
- Criteria for exclusion/inclusion of data should be clearly stated
- High-throughput sequence data should be uploaded before submission, with a private link for reviewers provided (these are available from both GEO and ArrayExpress)

Please outline where this information can be found within the submission (e.g., sections or figure legends), or explain why this information doesn't apply to your submission:

This information can be found in the Material and Methods, subsection Experimental Design. Number of replicates and exclusion criteria are outlined for each experiment. No high-throughput sequencing is included in the data.

Statistical reporting

- Statistical analysis methods should be described and justified
- Raw data should be presented in figures whenever informative to do so (typically when N per group is less than 10)
- For each experiment, you should identify the statistical tests used, exact values of N, definitions of center, methods of multiple test correction, and dispersion and precision measures (e.g., mean, median, SD, SEM, confidence intervals; and, for the major substantive results, a measure of effect size (e.g., Pearson's r, Cohen's d)
- Report exact p-values wherever possible alongside the summary statistics and 95% confidence intervals. These should be reported for all key questions and not only when the p-value is less than 0.05.

Please outline where this information can be found within the submission (e.g., sections or figure legends), or explain why this information doesn't apply to your submission:

Statistical tests used and N numbers are in the Figure Legends for each graph. p-values of less than 0.05 are included on the graph in the figures. p-values of greater than 0.05 are not shown. Exact p-values for all comparisons can be found in the source data for each graph. Explanation of statistical tests used can be found in the materials and methods, subsection Statistics.

For the presented generalized linear model, a Bayesian model formulation is used. Therefore Bayesian credible intervals are reported to support conclusions rather than p-values.

(For large datasets, or papers with a very large number of statistical tests, you may upload a single table file with tests, Ns, etc., with reference to sections in the manuscript.)

Group allocation

- Indicate how samples were allocated into experimental groups (in the case of clinical studies, please specify allocation to treatment method); if randomization was used, please also state if restricted randomization was applied
- Indicate if masking was used during group allocation, data collection and/or data analysis

Please outline where this information can be found within the submission (e.g., sections or figure legends), or explain why this information doesn't apply to your submission:

Samples were allocated based on treatments into groups (ie nocodazole, taxol or DMSO treatment and low or high glucose treatment). Masking was used prior to data analysis of secretion events. Information can be found in the Materials and Methods, subsection Experimental Design.

Additional data files ("source data")

- We encourage you to upload relevant additional data files, such as numerical data that are represented as a graph in a figure, or as a summary table
- Where provided, these should be in the most useful format, and they can be uploaded as "Source data" files linked to a main figure or table
- Include model definition files including the full list of parameters used
- Include code used for data analysis (e.g., R, MatLab)
- Avoid stating that data files are "available upon request"

Please indicate the figures or tables for which source data files have been provided:

Figure 1 and Figure 1 supplement- numerical and statistical data is provided.
Figure 2 and Figure 2 supplement- Matlab scripts used to analyze data, raw output data from Matlab scripts and numerical and statistical data are provided.
Figure 3- Matlab script to determine nearest neighbor distance and numerical and statistical data is provided. Matlab script to determine cluster is included in Figure 2 source data.
Figure 4- Numerical and statistical data is provided.
Figure 5- Numerical and statistical data for 5A is provided. Python scripts that produce the statistical analysis and plots for Figure 5 are provided.
Figure 6 and Figure 6 supplement- Numerical and statistical data is provided.
Figure 7 and Figure 7 supplement- Numerical and statistical data is provided.
Figure 8 and Figure 8 supplement- Numerical and statistical data is provided.
